# Sustained Release of Lidocaine from Solvent-Free Biodegradable Poly[(d,l)-Lactide-co-Glycolide] (PLGA): *In Vitro* and *In Vivo* Study

**DOI:** 10.3390/ma7096660

**Published:** 2014-09-16

**Authors:** Yi-Chuan Kau, Chia-Chih Liao, Ying-Chi Chen, Shih-Jung Liu

**Affiliations:** 1 Department of Anesthesiology, Chang Gung Memorial Hospital, Tao-Yuan 333, Taiwan; E-Mails: yichuan@cgmh.org.tw (Y.-C.K.); yichuan@cgmh.org.tw (C.-C.L.); 2 Graduate Institute of Medical Mechatronics, Chang Gung University, Tao-Yuan 333, Taiwan; E-Mail: yingtzai@gmail.com; 3 Department of Mechanical Engineering, Chang Gung University, Tao-Yuan 333, Taiwan

**Keywords:** poly[(d,l)-lactide-co-glycolide] (PLGA), lidocaine, sustained release, biodegradable pellets

## Abstract

Local anesthetics are commonly used for pain relief by regional nerve blocking. In this study, we fabricated solvent-free biodegradable pellets to extend the duration of lidocaine release without any significant local or systemic toxicity levels. To manufacture the pellets, poly[(d,l)-lactide-co-glycolide] (PLGA) was first pre-mixed with lidocaine powder into different ratios. The powder mixture was then compressed with a mold (diameter of 1, 5, 8 or 10 mm) and sintered at 65 °C to form pellets. The *in vitro* release study showed that the lidocaine/PLGA pellets exhibited a tri-phase release behavior (a burst, a diffusion-controlled release and a degradation-dominated release) and reached completion around day 28. Scanning electron microscope (SEM) photos show that small channels could be found on the surfaces of the pellets on day 2. Furthermore, the polymer matrix swelled and fell apart on day 7, while the pellets became viscous after 10 days of *in vitro* elution. Perineural administration of the lidocaine/PLGA pellets produced anti-hypersensitivity effects lasting for at least 24 h in rats, significant when compared to the control group (a pure PLGA was pellet administered). In addition, no inflammation was detected within the nerve and in the neighboring muscle by histopathology.

## 1. Introduction

Acute pain relief is an important issue for post-operative management. A meta-analysis covering approximately 20,000 patients and 800 publications revealed that 41% of all surgical patients experience moderate to severe acute postoperative pain [1]. Neuronal block with local anesthetics is one of the most effective methods to provide dynamic pain relief after major surgical procedures. Most local anesthetics bind to sodium channels in the nerve membrane, preventing channel activation and the large transient sodium influx associated with membrane depolarization. The excitation impulse therefore cannot be conducted along a nerve axon to cerebral cortex; thus, the sensation of pain will not be interpreted [2].

Single doses of local anesthetics, such as lidocaine, bupivacaine or ropivacaine, only provide analgesia for fewer than 8 h [3]. In order to prolong the period of drug release, a catheter is usually deployed in the epidural space or at the peripheral nerves for continuous infusion. Nevertheless, the continuous infusion of local anesthetics may cause complications, such as infection, catheter misplacement or systemic toxicity [4]. The development of a sustainable anesthetic delivery system can overcome these problems. An ideal drug delivery system for local anesthetic should: (1) achieve an adequate drug concentration at the target site; (2) provide a slow and constant release of the drug over a prolonged period; and (3) be biodegradable, so that a second operation to remove the delivery vehicle is not necessary [5].

In the last two decades, long-acting local anesthetics have been investigated in the forms of microspheres, liposomes, micro-needles and solid vehicles. Microspheres refer to spherical particulates, with diameters in the range of 50 nm to 2 mm. The source of the material can be divided into the categories of natural substances (such as dextran) and synthetic substances (such as poly(lactic-co-glycolic) acid (PLGA) or polylactic acid (PLA). Microspheres are widely used in biomedical aspects, such as diagnostics, immunoassays, cell separations, drug deliveries, *etc*. [6]. Curley *et al*. [7] published a series of studies on bupivacaine-polymer microspheres. They prepared microspheres using polylactic-co-glycolic acid polymers loaded with 75% w/w bupivacaine by a solvent evaporation method. The results showed that microspheres could produce prolonged blockade that ranged from 10 h to 5.5 days at the sciatic nerve of rats.

Liposomes are vesicles composed of an artificial lipid bilayer. The liposomes can be used as a vehicle of nutrients and drug administration. They are often composed of phosphatidylcholine-enriched phospholipids and may also contain mixed lipid chains with surfactant properties, such as egg phosphatidylethanolamine [8]. Mowat *et al*. [9] reported that large unilamellar vesicles exhibiting a pH gradient could efficiently encapsulate bupivacaine. The sustained drug release provided by liposomes resulted in an increase in the duration of nerve blockade as much as three-fold, compared to the duration provided via free drug. Microneedles are minimally invasive micron-scale needles protruding perpendicularly from a laterally-mounted platform. It is a painless method of micro-injection for not hitting pain receptors concentrated in the dermal layer of skin [10]. They are used for the delivery of local anesthesia, such as lidocaine, to lacerated skin regions in aiding the optimization of skin permeation kinetics [11].

In recent years, other types of solid vehicles containing lidocaine were also developed. Wang *et al*. [12] published a series of “Xybrex” studies. Xybrex is an implantable, malleable (with a consistency similar to modeling clay) and absorbable matrix containing 16% lidocaine (w/w). It can provide several days of functional sciatic nerve blockade. Tobe *et al*. [13] created a novel slow-release lidocaine sheet (SRLS) (30% lidocaine, w/w) with polylactic-co-glycolic acid. The SRLS continuously released lidocaine *in vitro* and inhibited hyperalgesia and c-fos expression in the spinal cord dorsal horn for one week. However, all of the previously mentioned drug delivery systems used solvents in the manufacturing process. The residual solvent may induce inflammatory reactions and has safety concerns when applied to human bodies [14].

The sciatic nerve supplies motor and sensory innervation to the posterior aspect of the thigh, as well as the entire lower leg. The block of the sciatic nerve can provide postoperative pain relief after lower limb surgery. Sciatic nerve blocks require an adequate set-up, because this large nerve resists local anesthetic penetration, leading to longer block onset time. For complete anesthesia of the leg below the knee, the saphenous nerve must also be blocked, either directly or via a femoral nerve block.

In this study, we employed a solvent-free heat compressing technique [15,16,17] to manufacture biodegradable lidocaine-loaded pellets that provide sustained release of lidocaine for postoperative pain relief after below knee amputation, knee replacement, foot and ankle surgery. The *in vitro* and *in vivo* release characteristics of pharmaceuticals from the pellets were investigated. In addition, the effect of biodegradable lidocaine-loaded pellets on the behaviors of rats with incision wounds was examined. Histological examination of the sciatic nerves and surrounding muscles was also completed.

## 2. Materials and Methods

### 2.1. Materials

The polymers used were poly[(d,l)-lactide-co-glycolide] (PLGA) with two different lactide:glycolide ratios of 50:50 (RESOMER^®^ RG 503) and 75:25 (RESOMER^®^ RG 756), both purchased from Sigma-Aldrich, St. Louis, MO, USA. The polymers were available in powder form with particle sizes ranging from 100 to 200 μm. A DuPont model TA-2000 differential scanning calorimeter was used to characterize the thermal properties of the polymer. The measured results suggested that the polymers’ glass transition temperature was in the range of 45–50 °C. The local anesthetic used was commercial-grade lidocaine hydrochloride powder with a particle size of 100 μm (Sigma-Aldrich, St. Louis, MO, USA).

### 2.2. Preparation of Lidocaine/PLGA Pellets

**Figure 1:**
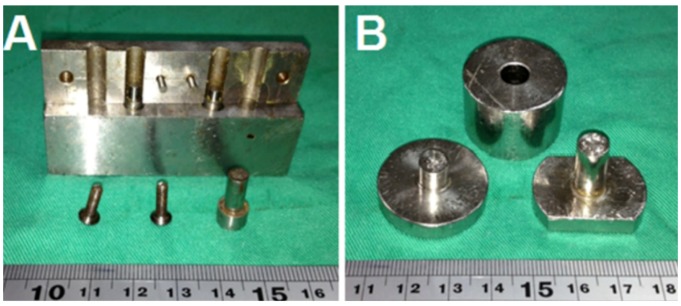
Molds with diameters of (**A**) 1 mm and (**B**) 8 mm. (Diameters of 5, 8 and 10 mm molds have the same geometric view.)

To fabricate the biodegradable pellets, PLGA were pre-mixed with lidocaine powder into different ratios (1:3, 1:5 and 1:10, respectively, for lidocaine to polymer weight ratios). The mixture was compressed into pellets with a mold (diameters of 1, 5, 8 or 10 mm), as shown in Figure 1.

**Figure 2:**
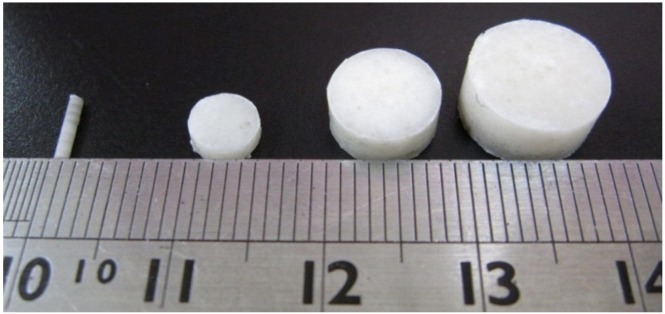
Photo of the manufactured biodegradable lidocaine/poly[(d,l)-lactide-co-glycolide] (PLGA) pellets.

The compressed pellets with the mold were then placed in an oven for sintering. The sintering temperature was set at 65 °C, which was higher than the polymers’ glass transition temperatures, but low enough to avoid destroying the lidocaine. The compression pressure was set at 90 MPa. The sintering time was 2 h in order to attain an isothermal sintering of the pellets. After sintering, drug-eluting implants of various sizes and geometries were then obtained, as shown in Figure 2 and Table 1 and Table 2.

**Table 1:** Dimensions and compositions of the lidocaine/PLGA pellets of various polymer-to-drug ratios.

Diameter (mm)	Polymer-to-drug ratio	Weight of polymer (mg)	Weight of anesthetic (mg)
1	3:1	4.86	1.62
1	5:1	5.4	1.08
1	10:1	5.89	0.59

**Table 2:** Dimensions and compositions of the lidocaine/PLGA pellets of different sizes.

Diameter (mm)	Polymer-to-drug ratio	Weight of polymer (mg)	Weight of anesthetic (mg)
1	5:1	5.4	1.08
5	5:1	50	10
8	5:1	200	40
10	5:1	400	80

### 2.3. In Vitro Lidocaine Elution Study

An *in vitro* elution method was employed to determine the release characteristics of lidocaine from the pellets. The pellets were placed in glass test tubes with 4 mL of phosphate buffer (0.15 mol/L, pH 7.4). All tubes were incubated under static conditions at 37 °C. The dissolution medium was collected for subsequent analyses after 24 h. Fresh phosphate buffer (4 mL) was then added for the next 24-h period, and this process was repeated over a 30-day period.

### 2.4. HPLC Analysis

The lidocaine concentrations in the buffer for the elution studies were determined by an HPLC assay standard curve [18]. The HPLC analyses were conducted on a Hitachi L-2200 Multisolvent Delivery System (Tokyo, Japan). The column used for the separation of lidocaine was an Atlantis C18, 4.6 × 150 mm, 5 μm HPLC column (Waters Corp., Milford, MA, USA). The mobile phase contained methanol and 10 mM ammonium formate (HCOONH_4_) (80/20, v/v). The absorbency was monitored at 210 nm, and the flow rate was 1.0 mL/min. All samples were assayed in triplicate, and sample dilutions were performed to bring the unknown concentrations into the range of the assay standard curve. A calibration curve was made for each set of the measurements (correlation coefficient > 0.99). The elution product can be specifically identified and quantified with high sensitivity using the HPLC system.

### 2.5. Subfascial Sciatic Nerve Implantations

The animal experimental protocol was approved by the Animal Care and Use Committee of Chang Gung University, Taiwan. All studied animals were cared for in accordance with the regulations of the National Institute of Health of Taiwan under the supervision of a licensed veterinarian. A total of 75 male Sprague-Dawley rats, weighing approximately 300 g each, were used. All animals were caged in an environment maintained with controlled relative humidity (20%–30%) at room temperature (24 °C), a 12 h light/dark cycle (lights on from 7:00 to 19:00) and free access to food and water. In addition, the rats were allowed to familiarize themselves with the experimental environment before the procedures began. Three rats were excluded from the study, due to accidental deaths. Data from 72 rats were included in the analysis.

**Figure 3:**
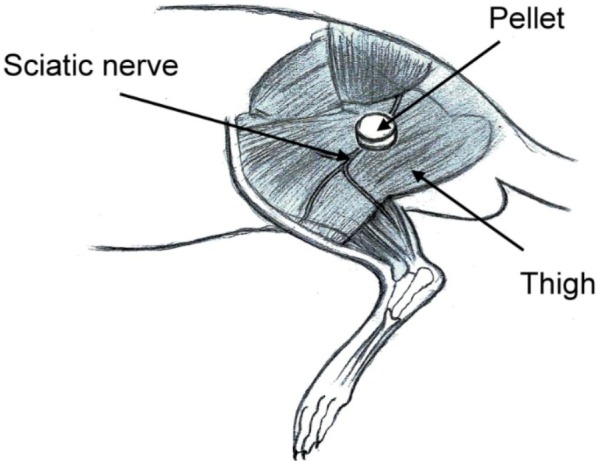
Schematic of the implantation of the lidocaine/PLGA pellet.

Eight millimeter-diameter lidocaine-loaded PLGA pellets (40 mg of lidocaine plus 200 mg of 50:50 PLGA) and pure PLGA pellets (240 mg of only 50:50 PLGA) were used for *in vivo* implantation. Six rats were used in each experimental group. Rats were anesthetized initially by 2% sevoflurane inhalation. A lateral incision of the left thigh was then made, and the sciatic nerve was exposed by a blunt division of the subcutaneous tissue and biceps femoris muscle. The sciatic nerve was freed from the epineurial fascia. A lidocaine/PLGA pellet or virgin PLGA pellet was applied underneath the fascia next to the sciatic nerve. After implantation, the superficial muscle layer and the skin incision were closed with 4-0 Vicryl sutures and 4-0 Prolene sutures sequentially (Figure 3).

### 2.6. Post-Operative Pain Model

**Figure 4:**
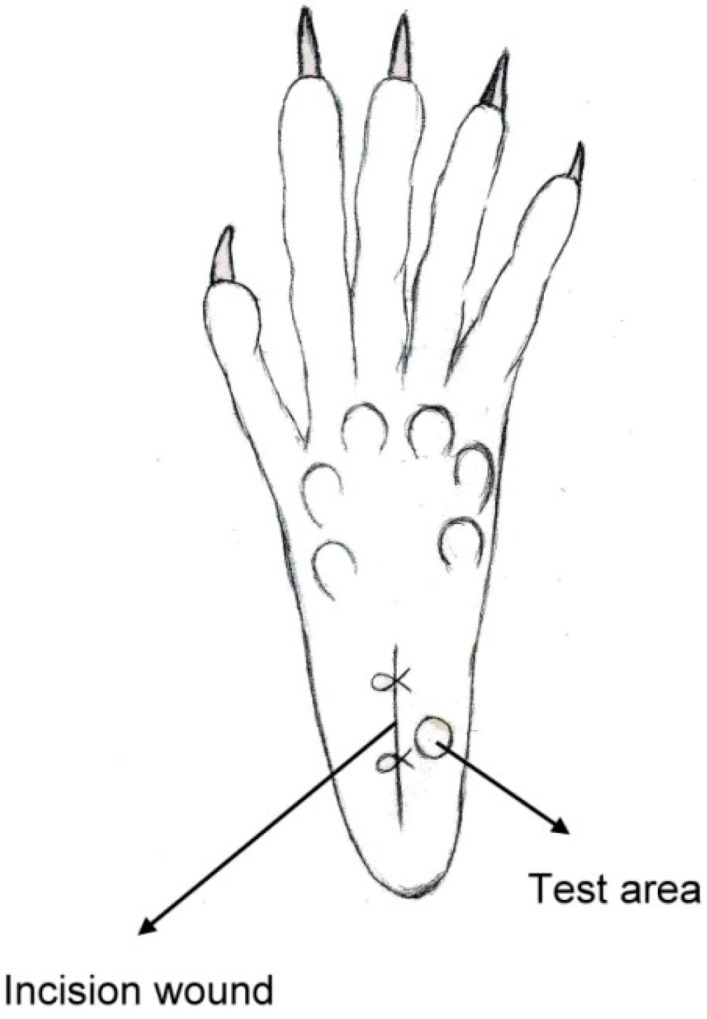
Schematic of the paw incision.

The classic Brennan paw incision model was used [19]. Immediately after the sciatic nerve implantation, a 1 cm longitudinal incision was made with a No. 11 blade on the medial plantar surface of the left hind paw. The incision began 0.5 cm distal from the end of the heel to the first set of footpads. The skin, fascia and plantaris muscle were all elevated using forceps and incised longitudinally (Figure 4). The skin was closed with 5-0 nylon sutures, and intraperitoneal antibiotics were given. After surgery, the animals were allowed to recover from the anesthesia in their cages. Wounds were checked for whether or not there was dehiscence before each behavioral examination.

### 2.7. Behavioral Examination

Rats were isolated in individual plastic chambers with a stainless steel mesh floor and were allowed 15 min to acclimate to the environment. The mechanical withdrawal threshold was assessed using von Frey filaments (Touch-Test Sensory Evaluators; Stoelting Co., Wood Dale, IL, USA). The filaments were applied vertically to an area next to the incision wound of the plantar surface for 5 s with gentle bending of the filament. The positive response was scored if the hind paw was withdrawn. The tactile stimulus producing a 50% likelihood of withdrawal threshold was determined using the Dixon up-down method [20].

### 2.8. In Vivo Lidocaine Release Study

The concentrations of lidocaine in local tissues were measured daily for 10 days after the administration. Six rats were euthanized with an intraperitoneal injection of sodium pentobarbital (70 mg/kg) for each day. After opening the wound and removing the pellet at the left thigh, the sciatic nerve and surrounding muscle were sampled at 0.05 g. Interstitial fluid was tapped via micropipe from exudate around the sciatic nerve. All tapping samples and fluid were assayed after dilution with phosphate buffered saline and were characterized by the HPLC assay according to the standard curve.

### 2.9. Surface Morphology

The surface morphological variation of the lidocaine/PLGA pellets during *in vitro* and *in vivo* degradation was observed under scanning electron microscope (SEM) (Hitachi S-3000N, Tokyo, Japan) for 10 days. Prior to examination, the surfaces were covered with a thin layer (60 Å in thickness) of gold to make them conductive. The voltage used was 10 kV.

### 2.10. Cytotoxicity of Lidocaine/PLGA Pellets

The cytotoxicity of lidocaine/PLGA pellets was checked by the MTT assay (Roche, Grenzach-Wyhlen, Germany) of cell viability. The basic principle is that MTT (3-(4,5-dimethylthiazol-2-yl)-2,5-diphenyltetrazolium bromide), a yellow tetrazole, is used. In living cells, the tetrazolium ring is cut by the mitochondrial dehydrogenase, and the color changes from light yellow to purple when forming formazan, which is visible under the microscope when viewing formazan crystals. If cells die, mitochondrial dehydrogenation will not be activated, so the crystals remain yellow. Tetrazolium dye assays can also be used to measure cytotoxicity (loss of viable cells) or cytostatic activity (shift from proliferative to resting status) of potential medicinal agents and toxic materials [21].

In our study, 1 mL of pellet eluting solution was collected at 24-h intervals for 10 days. From 5,000–10,000 cells of the human fibroblast suspension were seeded in a 96-well tissue culture plate (Falcon, Becton-Dickinson, Oxnard, CA, USA). After a 24 h incubation period, the solutions in the wells were replaced by the pellets’ eluting solutions for another 24 h. Five milligrams of MTT powder (Sigma Chemical Co., St. Louis, MO, USA) were dissolved in 10 mL of second-distilled water to get the MTT solution. After incubation, 20 μL of MTT solution were added to each well for 4 h. This was followed by the addition of 200 μL of 0.04 N HCl in isopropanol. The enzyme immunoassay analyzer automatically interpreted within a wavelength of 490–630 nm and measured the absorbance (OD). The percent of cell survival is expressed as relative survival index (RI%) = (OD sample/OD control) × 100%.

### 2.11. Histopathology

We excised the sciatic nerves and surrounding muscles on days 1, 4, 7 and 10 after the pellet implantation. The rats were euthanized with an intraperitoneal injection of sodium pentobarbital (70 mg/kg). The sciatic nerve and surrounding muscle were sampled and fixed overnight in 10% buffered formalin phosphate. The tissue was then cut and stained with hematoxylin and eosin stain. The slides were checked for the presence of inflammatory reaction.

### 2.12. Statistical Analysis

Data were collected from hexaplicate samples and shown as the mean ± standard error. Statistical analysis was conducted using SPSS (Version 17.0; IBM Inc., Armonk, NY, USA). Behavioral studies were analyzed using one-way ANOVA followed by a Student–Newman–Keuls *post hoc* test. Differences were considered statistically significant for *p*-values <0.05.

## 3. Results and Discussion

### 3.1. In Vitro Release Characteristics of Lidocaine from Biodegradable Pellets

During the processing of polymer pellets, the formation of a homogeneous melt from powder particles involved two steps. (1) Sintering: the polymeric particles stick or fuse together at their points of contact around the anesthetic particles. This fusion zone grows, until the mass becomes a three-dimensional network, with relatively little density change; (2) Densification: at some point in the fusion process, the network begins to collapse into the void spaces between the polymer and the drug. These spaces are filled with molten polymer that is drawn into the region by capillary forces [22]. The drug is then encapsulated by the polymer to form a composite pellet.

**Figure 5:**
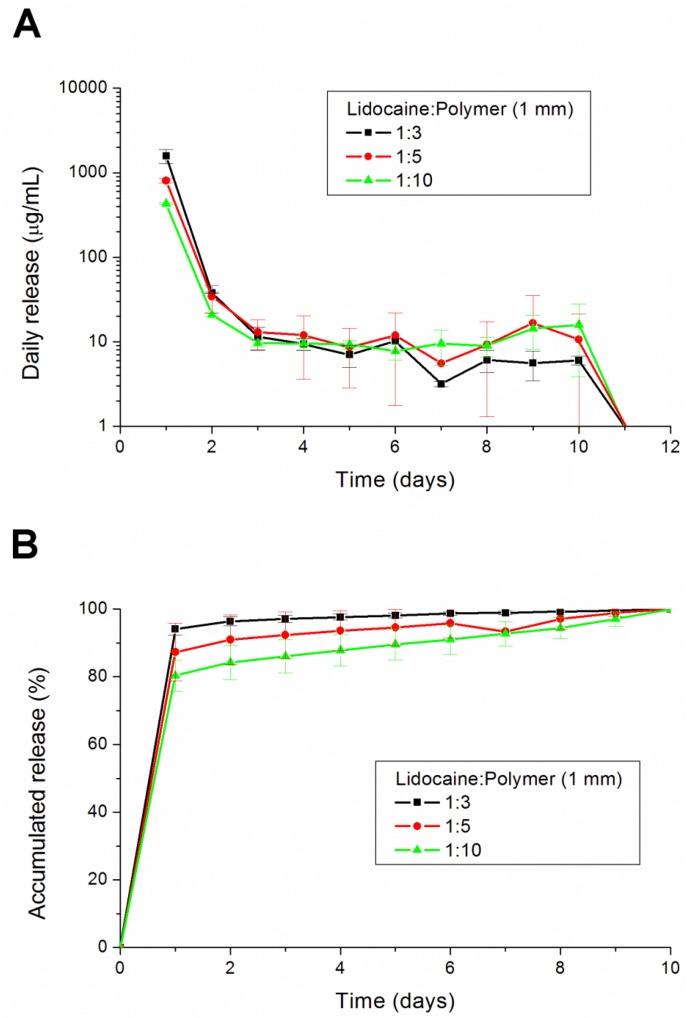
The (**A**) daily and (**B**) accumulated release kinetics of the three different ratios (1:3, 1:5 and 1:10) of the lidocaine-polymer mixture.

Three different ratios (1:3, 1:5 and 1:10) of lidocaine-polymer mixture were compared in the study. The results in Figure 5 show that the 1:3 group had a lower drug concentration than the other two groups after 6 days. When the lidocaine loading is high, the drug particles cannot be completely encapsulated and protected by the polymer, and then, they quickly dissolved. On the other hand, with low levels of anesthesia, the anesthetic particles can be separated in the polymer matrix. With an increase in the amount of anesthetic, anesthetic particles will be connected together to form a channel. This anesthetic will be released by channel diffusion [23]. The burst release phenomenon cannot be diminished by increasing the volume of the polymer matrix.

**Figure 6:**
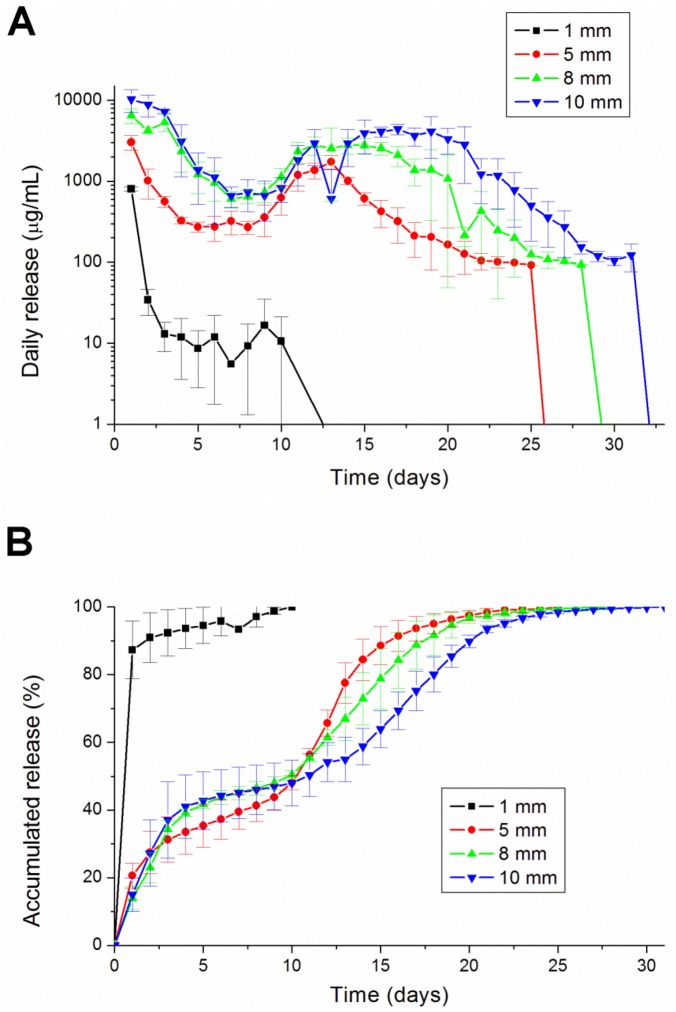
The (**A**) daily and (**B**) accumulated release kinetics of the four different diameters (1, 5, 8 and 10 mm) of the lidocaine-polymer mixtureusing PLGA 503 polymers.

Comparing the different diameters of lidocaine/PLGA pellets, we found that the larger the diameter, the longer the duration of lidocaine release (Figure 6). The 1 mm-diameter pellet was too small to fully encapsulate the lidocaine and then completely degraded after 10 days. The other three sizes of pellets could maintain a sustainable release for more than 25 days. Besides the 1 mm-diameter pellet, the lidocaine showed a tri-phase release pattern, with an initial burst phase on day 1, followed by a more gradual and sustained release of the drug until day 8, where a second peak of drug release was observed from days 8 to 17. The concentration gradually decreased thereafter.

**Figure 7:**
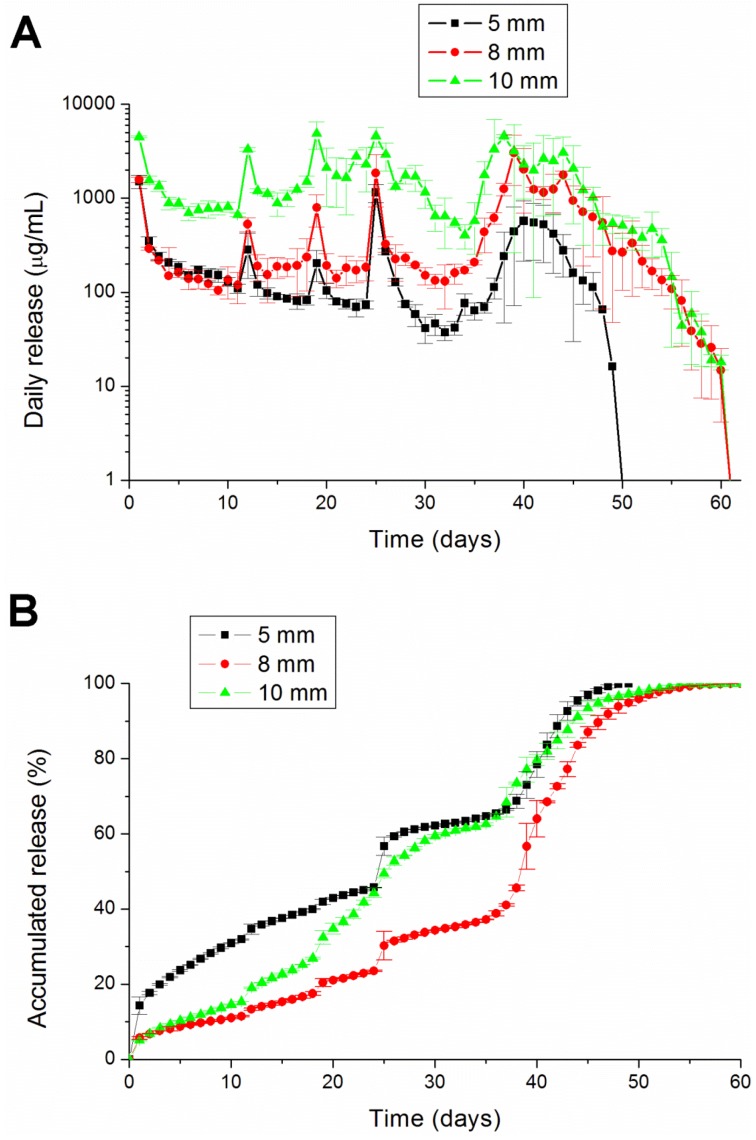
The (**A**) daily and (**B**) accumulated release kinetics of the three different diameters (5, 8 and 10 mm) of the lidocaine-polymer mixture using PLGA 756 polymers.

The degradation rate can be varied with the percentage of glycolic acid in the copolymer. The higher the ratio of the lactic acid, the longer the dissolution time was for the PLGA [24]. PLGA 756 contains more lactic acid than PLGA 530 (75% *vs.* 50%). Therefore, PLGA 756 can retain analgesics in the pellets longer than PLGA 503 can (Figure 7).

### 3.2. In Vivo Lidocaine Release Study

**Figure 8:**
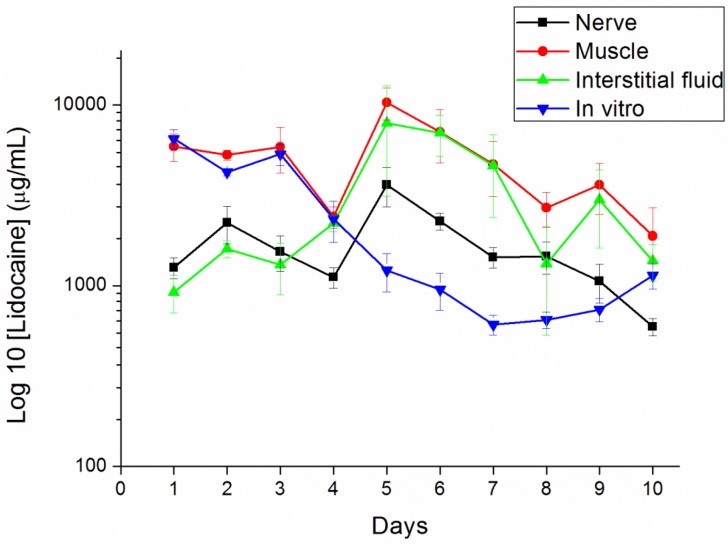
*In vivo* release characteristics of the lidocaine/PLGA pellets in sciatic nerve, muscle, interstitial fluid and *in vitro*.

*In vivo* drug levels were measured for 10 days using the HPLC assay. We euthanized six rats every day to examine the lidocaine concentrations. No migration of the pellets away from the implantation site proximal to the sciatic nerve was observed in any rats. The measured concentrations of lidocaine are shown in Figure 8. The concentration of lidocaine at the observed time points peaked on day 5 and decreased slowly after. The peak time points were faster in *in vivo* than *in vitro* elution (day 13; shown in Figure 6). This may be caused by enzyme degradation [25,26] and cellular activity [24] in the animals’ bodies. The potential autocatalytic degradation of PLGA with local build-up of degradation products may also contribute to the acceleration of the degradation process. The lidocaine concentrations in the muscle and interstitial fluid were even higher than in the sciatic nerve. The results implied that the implantation site received more of the analgesic effect than the ipsilateral paw did.

### 3.3. Behavioral Examination

**Figure 9:**
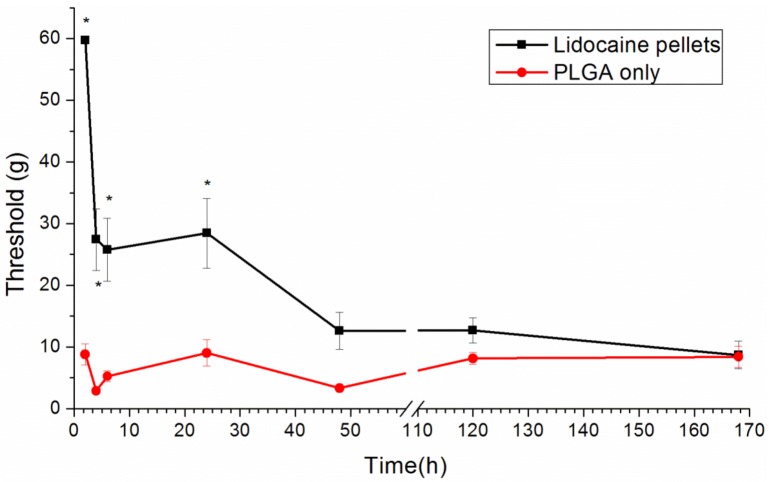
The effect of the lidocaine/PLGA pellet application in the rat model of post-operative pain. A lidocaine/PLGA pellet (lidocaine 40 mg plus PLGA(503) 200 mg) or a PLGA pellet (PLGA(503) 240mg without lidocaine; control) was applied perineurally to the sciatic nerve. The hind paw withdrawal thresholds (g) are shown as the mean ± standard error (n = 6 in each group). * *p* < 0.05 compared with the control group.

We used a post-operative pain model for the pain produced by the paw incision. Paw incision pain can be indicated by a reduced paw withdrawal threshold [19]. The paw withdrawal threshold in the lidocaine-implanted group significantly increased from 2 to 24 h after the paw incision (*p* < 0.01, by one-way ANOVA), slightly increased from days 2 to 5 after the paw incision and, finally, dropped to the baseline, the same as those in the control group (Figure 9).

When comparing the *in vivo* release study and the behavioral exam, we did not find any significant anti-hypersensitivity effects on day 5. This could be explained by the limitation of Brennan’s model. In this model, the significantly reliable and quantifiable mechanical hyperalgesia lasts for 2–3 days after surgery [19]. Clinically, patients suffer from maximal pain around 12 h after surgery and need more potent analgesics for only three days [27]. Therefore, the analgesic effect on day 5 is not important.

**Figure 10:**
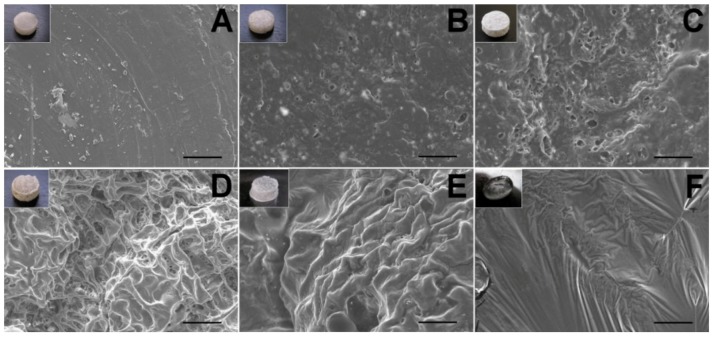
The SEM micrographs of lidocaine/PLGA pellets during *in vitro* degradation ((**A**) day 0; (**B)** day 1; (**C**) day 2; (**D**) day 4; (**E**) day 7; (**F**) day 10). Scale bar = 50 μm. Photographs in the upper left corners are the gross appearance of various pellets.

### 3.4. Surface Morphology

The surface changes during the *in vitro* experiment are shown in Figure 10. For a water-soluble local anesthetic in a hydrophobic polylactide matrix, the release mechanisms are controlled by channel diffusion, osmotic pressure and polymer degradation [28]. On the first day, the lidocaine, which was incompletely encapsulated by the polymer on the surface, dissolved quickly to form many holes and caused the burst release phenomenon. From day 2 to day 7, the presence of surface orifices and channels in the pellet accelerated the diffusion, as well as the anesthetic release. Water was taken up by the water-soluble drug with high osmotic pressure through the polymer, causing swelling of the particle. The polymer matrix might have broken under this swelling to form openings for drug release. On day 10, the pellet gradually dissolved, and the drug release was combined with polymer degradation [23].

**Figure 11:**
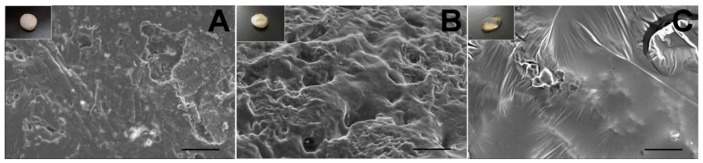
The SEM micrographs of the lidocaine/PLGA pellets during *in vivo* degradation ((**A**) day 2; (**B**) day 6; (**C**) day 8). Scale bar = 50 μm. Photographs in the upper left corners are the gross appearance of various pellets.

Comparing the *in vitro* and *in vivo* morphology (Figure 11), we found that the pellet dissolved and degraded faster *in vivo* than *in vitro* (day 8 *vs.* day 10). The result was consistent with the release studies.

### 3.5. Cytotoxicity of Lidocaine/PLGA Pellets

**Figure 12:**
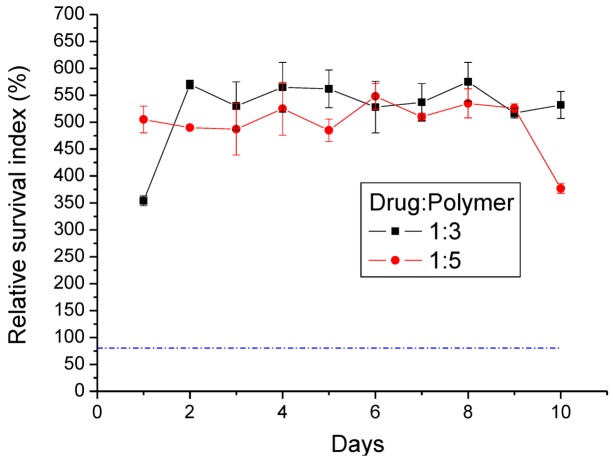
Relative survival index (RI%) of two different ratios of the lidocaine/PLGA pellets (the dashed line is the 80% RI ratio).

The MTT results showed that the average relative survival index (RI) ratio of the lidocaine/PLGA pellets was higher than 80% (dashed line in Figure 12). This implied that lidocaine could promote cell viability, mainly attributable to the fact that lidocaine plays the role of membrane stabilizer and phospholipase A2 inhibitor [29].

### 3.6. Histopathology

**Figure 13:**
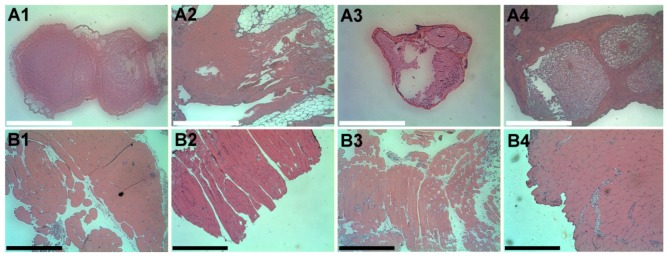
Histological images of the cross-section of the sciatic nerve (**A1**–**A4**) and surrounding muscle (**B1**–**B4**) on days 1, 4, 7 and 10, respectively, after the lidocaine/PLGA pellets were implanted. There was no accumulation of plasma and leukocytes, especially granulocytes. Scale bar = 5 μm.

The cross-sections of the sciatic nerve and muscles around the site of drug administration at each time-point (on days 1, 4, 7 and 10) are displayed in Figure 13. Neither inflammation (which was indicated by the increased infiltrating cells, such as macrophages, giant cells of the foreign body type, lymphocytes and plasma cells) nor tissue necrosis was observed in the histological examination.

## 4. Conclusions

This paper has explored compression sintering techniques to manufacture biodegradable polymer pellets for long-term drug release. We evaluated the pellets by an elution method and an HPLC assay. The effect of the different processing parameters on the release characteristics of the pellets was investigated. The total effective release period of lidocaine from the pellets could be prolonged by using lower anesthetic-to-polymer ratios, increasing the diameter or increasing the ratio of lactic acid in the PLGA polymer. The slow-release pellet could release lidocaine over 25 days and enhance post-operative pain management for at least 24 h. It had the advantage of not only producing long-term post-operative analgesic effects, but also preventing inflammation induced by high local concentrations of lidocaine.

Further studies can investigate the biodegradable pellets in different animal pain models, such as neuropathic pain or the inflammatory pain model. The other medial duration of anesthetics should also be investigated. Eventually, biodegradable local anesthetic pellets may be used in humans for the treatment of various pain symptoms.
